# Supervisor-Subordinate Age Dissimilarity and Its Impact on Supervisory Ratings of Employability: Does Supportive Learning Context Make a Difference?

**DOI:** 10.3389/fpsyg.2021.763746

**Published:** 2021-12-16

**Authors:** Dora Scholarios, Beatrice Van der Heijden

**Affiliations:** ^1^Department of Work, Employment and Organisation, University of Strathclyde, Glasgow, United Kingdom; ^2^Institute for Management Research, Radboud University Nijmegen, Nijmegen, Netherlands; ^3^Faculty of Management, Open Universiteit, Heerlen, Netherlands; ^4^Department of Marketing, Innovation and Organisation, Ghent University, Ghent, Belgium; ^5^Business School, Hubei University, Wuhan, China; ^6^Kingston Business School, Kingston University, Kingston Upon Thames, United Kingdom

**Keywords:** age stereotyping, status incongruence, relational demography, employee-supervisor dyad, employability, learning opportunities at work

## Abstract

Status incongruence resulting from a supervisor who is younger than their subordinate potentially leads to age stereotyping of employees. This article investigates the relationship between age difference and supervisory ratings of five competence-based measures of subordinate employability (Occupational Expertise, Anticipation/Optimisation, Personal Flexibility, Corporate Sense, and Balance). In addition, we consider the buffering role of a supportive learning context which allows older workers access to learning resources. Learning context is represented by duration of the supervisory relationship, perceived organizational learning climate and participation in, and application of, training and development. Using 295 dyads of employees and their direct supervisors in a Dutch building company, findings show that age dissimilarity reflecting status incongruence was related to lower supervisory ratings of Occupational Expertise (job-related competence) and Corporate Sense (social/organizational competence) regardless of learning context. Longer duration relationships exacerbated, rather than buffered, the age difference effect on some types of supervisory ratings. The implications of these findings for age stereotyping with regard to employability are considered.

## Introduction

The implications of age stereotyping for older workers’ career potential has received little attention so far (De Vos et al., 2020). Most research on older workers (variously considered as those from 40 to near retirement age) has centred on whether they are more or less competent than their younger colleagues, more expensive, treated with prejudice or require unique workplace interventions (Finkelstein and Farrell, 2007; Posthuma and Campion, 2009; Truxillo et al., 2015). Although the influence may be subtle, it is likely that age stereotypes lead supervisors to attach less value or recognition to their older subordinates (Van der Heijden et al., 2009). Either as a result of these perceptions, or as a self-fulfilling prophecy, highly qualified older employees may face progressively declining re-employability and barriers to career sustainability (Van der Heijden et al., 2020).

This article extends earlier studies on age stereotyping in evaluations which considered only the age of the ratee. Specifically, we examine whether age differences between employees and their supervisors, in particular, when supervisors are younger than their employees, influence supervisors’ employability ratings. We build on Tsui and O’Reilly’s (1989) concept of relational demography which proposes that the comparative (dis)similarity in demographic attributes of a superior and a subordinate may affect a range of work outcomes, in our case supervisors’ evaluations of their workers’ employability (see also Guillaume et al., 2012; Van der Heijden, 2018). To the best of our knowledge, the present empirical study is the first to shed much-needed light on the linkage between relational age and workers’ employability, incorporating several dimensions of employability. Building upon the sustainable careers paradigm (Van der Heijden and De Vos, 2015; De Vos et al., 2020), we argue that the age difference in the employee-supervisor dyadic relationship comprises an important contextual factor that influences one’s career potential. Ongoing demographic change (i.e., ageing and dejuvenization) of the workforce across the globe (Smith and Martin, 2018), urges career scholars to detect challenges related to these phenomena and evidence-based solutions aimed at preserving workforce strength throughout the life-span (Van der Heijden, 2018). Productivity, being one of the three key indicators of sustainable careers, next to health and happiness (Van der Heijden et al., 2005), is reflected in an employee’s current performance as well as his/her future career potential which are essential for the long-term performance of organizations (De Vos et al., 2020). As such, we argue that employability is a key performance indicator in contemporary working life.

While age diversity can give an organization a competitive advantage (Harvey, 2012; Burmeister et al., 2018), as individuals have a tendency to prefer homogeneous group settings, the effect of comparative (dis)similarity is dependent on how diversity can be harnessed for effectiveness at the workplace (Tsui et al., 1992). The relational approach to diversity focuses on the relationship between an individual’s characteristic, in our case the demographic of age, and the distribution of this characteristic in the individual’s unit (Riordan, 2000), in our case, the dyad of the employee and his/her supervisor. Accordingly, diversity refers to the degree of (dis)similarity or whether the employee’s age is shared by the supervisor in the specific dyad.

We also examine the role played by a supportive learning context focusing on the duration of the working relationship between an employee and their supervisor, perceived organizational learning climate (OLC) and training/development practices. For the moderating effect of the duration of the working relationship, we borrow insights from Harrison et al. (2002) who found that over time, as people collaborate, they have more opportunities for interaction and exchange of personal, idiosyncratic information. As a result, the impact of surface-level diversity (such as age) becomes less important in mutual evaluations. With regard to learning opportunities, building on the notion of situational strength (Meyer et al., 2010), we posit that OLC, and training and development practices are important situational factors to consider. Situational strength is defined as “implicit or explicit cues provided by external entities regarding the desirability of potential behaviors” (*ibid.*, p. 122), and is argued to result in psychological pressure, in our case on the employee, to engage in and/or refrain from particular courses of action, such as engaging in career development.

A strong learning climate, with appropriate training and development opportunities, reflects HRM practice which is essential for protecting and enhancing the sustainability of employees’ careers across the life-span. Subsequently, such situations will moderate individual difference–outcome relationships. In this study, we investigate whether the availability of learning opportunities influences the impact of age dissimilarity on employability ratings. More specifically, learning opportunities which target older workers influence perceived organizational support and career satisfaction, and ultimately may contribute to employees’ intention to remain (Armstrong-Stassen and Ursel, 2009). As employees age, however, managers are less actively engaged in their employees’ career development. Specifically, supervisors may assess a ‘pay-off’ period for training and career activities for older employees. Equally, older workers themselves assess whether the investment is worth the effort, resulting in a self-fulfilling prophecy in terms of supervisors’ evaluations (Van der Heijden and De Vos, 2015).

In this article, we first establish the value of considering age dissimilarity in supervisor-subordinate evaluations before presenting the case for a broad competence-based definition of employability in order to understand supervisors’ perceptions of older workers (Van der Heijde and Van der Heijden, 2006). We then formulate our research hypotheses which are tested in a study of supervisor-subordinate dyads in the Dutch workforce of a multinational company. The findings extend our understanding of age stereotyping in the workplace and of HRM practices aimed at enhancing older workers’ employability, particularly through the provision of learning opportunities.

## Theoretical Framework

### Supervisor-Subordinate Age Dissimilarity and Evaluations of Employability

Vertical dissimilarity refers to differences between supervisor and subordinate characteristics (Guillaume et al., 2012). The demographic characteristics of supervisor-subordinate dyads have been shown to negatively influence performance evaluations (Liden et al., 1996; Pearce and Xu, 2012). One explanation comes from the similarity-attraction paradigm which incorporates demographic variables, such as age, as well as attitudes (Riordan, 2000). The ‘similar-to-me’ hypothesis holds that people will be rated higher the more similar they are to the rater, or the more similar the rater believes people are to him or herself. Relational demography expands on similarity research by exploring the extent to which the comparative demographic characteristics of supervisor-subordinate dyads influence work outcomes, such as performance ratings (O’Reilly et al., 1989); e.g., through interpersonal attraction, based upon similarity in attitudes, values, and experiences or through frequency of interactions (Shore et al., 2003). In particular, supervisors and subordinates in the same age cohort tend to have common experiences (Lawrence, 1988; e.g., taking care of parents or losing close friends that pass away), and these experiences forge common values, and, in turn, smoother interactions (Geddes and Konrad, 2003).

In line with Tsui and O’Reilly (1989), we propose that these similarity effects account for variance beyond that accounted for by simple demographic attributes. Whether this is due to actual performance deficits or bias, such as age stereotyping, the effects are the same in that this relative age dissimilarity influences the career opportunities of older workers. We also propose that inconsistencies between a person’s relative status ranking on different status dimensions (e.g., age or organizational position) may, next to perceptions of similarities, also affect that person’s attitudes and behaviors (Collins et al., 2009). In particular, extending both relational demography and the idea of age norms, Tsui et al. (2002) argued that relational norms of age exist among supervisor-employee dyads, indicating that when employees are younger, less educated and have shorter tenure than their supervisors (i.e., when there is clear status incongruence), they receive higher performance ratings and vice versa (Yang and Matz-Costa, 2018). In other words, subordinates that report to a younger supervisor experience status incongruence and, subsequently, respond negatively, because of perceived violation of the career timetable associated with supervisory positions, or because of a lack of trust in their supervisor’s capacity to lead them adequately (Perry et al., 1999).

In support of this status incongruence effect, previous studies representing *directional* age differences have indicated that the age gap between a superior and a subordinate may be more problematic in one direction (when the superior is younger than the subordinate) than in the other (Tsui et al., 1995). As earlier scholarly work has failed to find an effect for *non-directional* age differences, where the supervisor may be either older or younger than the subordinate (Tsui and O’Reilly, 1989; Liden et al., 1996), following Perry et al. (1999), this article focuses on directional effects only. More specifically, it is not surprising that earlier work investigating non-directional age differences (wherein the absolute difference value or the square of the difference between supervisor and employee was used to measure relational age) did not find significant effects on work outcomes (e.g., Bakar and McCann, 2014) as this approach is problematic. The dissimilarity end of the continuum represents two different types of dyads (older supervisor-younger employee and younger supervisor-older employee dyads), herewith masking potentially important differences that can be ascribed to age-difference directionality. Relational norms theory (Tsui et al., 2002) suggests that a younger supervisor-older employee dyad reflects a relationship that violates traditional status norms and which is assumed to have an effect on performance ratings.

### Age Dissimilarity and Evaluations of Employability

We adopt the broad competence-based definition of employability provided by Van der Heijde and Van der Heijden (2006, p. 453): ‘the capacity of continuously fulfilling, acquiring or creating work through the optimal use of competences’. This definition acknowledges multiple facets of employability, including a behavioral tendency, internal and external labor market opportunities (Forrier and Sels, 2003), and the ability to identify and realize future career opportunities (Fugate et al., 2004). In addition to a domain-specific dimension of Occupational Expertise, Van der Heijde and Van der Heijden’s (2006) conceptualization proposed four generic competences: Anticipation/Optimization (preparing for and adapting to future changes in a personal and creative manner, and striving for the best possible results); Personal Flexibility (the capacity to easily adapt to all kinds of changes in the internal and external labor market beyond one’s immediate job); Corporate Sense (participation in different workgroups, including organizations, teams, occupational communities and other networks, which involves sharing responsibilities, knowledge, experiences, feelings, credits, failures, and goals); and Balance (compromise between opposing interests, such as one’s own opposing work, career, and private interests, and employer/employee interests). Our focus on supervisors’ evaluation of their employees’ career potential, based on multiple dimensions of employability (rather than occupational expertise alone; see Van der Heijden, 2018), is consistent with the emergence of a ‘life-long learning’ perspective of careers (De Vos et al., 2020). This perspective acknowledges that qualifications alone are insufficient for remaining competitive in current labor markets. In other words, contemporary labor markets require that employees and their supervisors both protect strong performance in their current job as well as further enhancing their employability or career potential in the future or in other jobs (Van der Heijde and Van der Heijden, 2006) in order to safeguard the sustainability of their career (*ibid.*).

In general, employers seem to believe that older workers perform better than younger ones in some ways, such as interpersonal skills (Loretto and White, 2006), but are more negative regarding other aspects of employability; e.g., flexibility, adaptability to technology, motivation for learning or training, and well-being (Warr, 2000). Research spanning decades has revealed the presence of such age stereotyping (e.g., Rosen and Jerdee, 1976; Kite and Johnson, 1988; Boerlijst et al., 1998; Chiu et al., 2001; Posthuma and Campion, 2009; Finkelstein et al., 2015; Lamont et al., 2015; Kulik et al., 2016; Zaniboni et al., 2019). As a consequence, managers may avoid hiring older workers or pay less attention to their career development (Mulders et al., 2018) in the belief that the ‘return on investment’ is likely to be small (Fleischmann et al., 2015). Given that this non-intervention begins at the age of 40 when most employees will be employed for another 25 or more years (assuming a retirement age ranging, for example, from 65–68 in Europe, 66–67 in the US, 63–65 in Japan and 62–65 in Singapore), such stereotyping is short-sighted. Moreover, employees themselves evaluate whether career investments are worthwhile; self-confidence for career-relevant learning and expertise development also decline with age (Simpson et al., 2002).

If both supervisors and employees are less actively engaged in skill enhancement, older employees are more likely to develop narrow expertise, which limits their employability. Supervisor willingness to invest in their employee also may decline, leading to a self-fulfilling prophecy with respect to supervisors’ evaluations (Van der Heijden et al., 2009). Thus, age dissimilarity between supervisors and employees may also create a supervisory relationship which itself is a source of age stereotyping and decision-making (Kunze and Menges, 2017).

We build on these arguments to formulate the following hypothesis:

*Hypothesis 1: The greater the directional age difference (younger supervisor-older employee dyad) between a supervisor and a subordinate, the lower will be the supervisor’s ratings of employability with respect to the subordinate’s: (a) Occupational Expertise, (b) Anticipation/Optimization, (c) Personal Flexibility, (d) Corporate Sense and (e) Balance*.

### Supportive Learning Context as a Moderator of Directional Age Difference Effects

Assuming older workers may struggle or lack the motivation to access developmental resources for building their career potential, a supportive learning environment will enhance their capacity to access further workplace resources (Hobfoll, 2011). Increased access to resources not only enhances actual employability (e.g., job expertise as well as knowledge of how they fit into the organization), it is also likely to reduce the effects of any age-related norms on evaluations of their employability. We focus on three learning context variables which could act as developmental resources for employees: duration of the supervisory relationship, perceptions of a positive OLC, and the individual’s participation in training and development and its application to their immediate job role.

#### Duration of the Working Relationship

Scholars have suggested that the duration of superior-subordinate interaction moderates the effects of demographic dissimilarity (Avery et al., 2012). Building on the notion of Harrison et al. (2002) who argued and found support for the role of time as a medium in collaborations, we assume that the impact of the demographic attribute of employee age (a surface-level attribute) on supervisor employability ratings becomes less important over time. In particular, in initial interactions, mutual evaluations are based on surface-level features (Berger et al., 1980; Schneider et al., 1995). However, over time, as an employee and his/her supervisor have had more time to interact and are enabled to observe larger samples of each other’s behaviors (Gruenfeld et al., 1996), more meaningful dyadic relationships will develop and age dissimilarity will become less salient.

Such arguments recognize that supervisor evaluations of their employees often occur under conditions of inadequate information, many distractions, and with minimum time and thought, thus opening the possibility of stereotyping (Smith et al., 2006). Where there is a lack of prior experience and individualized information, reliance on stereotypes is cognitively efficient. In the case of employees for whom the supervisor has sufficient or ample prior experience, the motivation to be cognitively efficient will still exist to the extent that there are time pressures and other task demands, but there is no need to rely on stereotypes to ‘fill in the blanks’. Alternatively, when there is a history of interaction, the supervisor may conclude that the employee is a well-known entity, and thus that there is less need to devote cognitive resources to the evaluation problem. Following this argument, we hypothesize the following:

*Hypothesis 2: A longer working relationship between a supervisor and subordinate will decrease the strength of the negative relationship between supervisor-subordinate directional age difference (younger supervisor rating older subordinate) and supervisory employability ratings with respect to the subordinate’s: (a) Occupational Expertise, (b) Anticipation/Optimization, (c) Personal Flexibility, (d) Corporate Sense and (e) Balance*.

#### Employee Perception of Organizational Learning Climate

Further development of employability can only be attained if employees are provided with relevant and frequent learning experiences (Gerken et al., 2016; Van der Heijde et al., 2018). For older workers, HRM policies and practices which encourage development are particularly important (Pak et al., 2019), acting as a signal that they are valued, enhancing their career satisfaction and intention to remain (Armstrong-Stassen and Ursel, 2009).

Previous research has identified OLC as a predictor of various employee behaviors demonstrating employability competences; e.g., asking for feedback and reflection, or sharing one’s knowledge and experiences (Kyndt et al., 2018). OLC may be especially critical for older workers’ employability (Pak et al., 2019). Opportunities to develop by learning from expert colleagues (Liu, 2018) and team style (Van der Heijden et al., 2005; Van der Heijde et al., 2018) have been shown to stimulate learning, personal flexibility, the development of individual competencies (Van der Heijden et al., 2009), and attitudes such as organizational commitment (Govaerts et al., 2011). We might also expect workers who have enjoyed a supportive climate to be in more appropriate careers (Eldor and Harpaz, 2016) and higher occupational levels with more job control (Edwards et al., 2006). This should, in turn, be reflected in higher employability ratings. We argue that a supportive OLC is especially important in cases of large directional supervisor-subordinate age differences. With greater age dissimilarity, supervisors may be less attuned to the distinctive developmental preferences of older employees, such as, a preference for intrinsically rewarding development or social contact rather than promotion opportunities (Kooij et al., 2011). More visible organizational options for career development provide situational cues to help older employees - and indeed their younger supervisors - craft appropriate strategies, recognizing that there may be fewer personal resources or opportunities (e.g., promotion) available to aging employees (Kanfer and Ackerman, 2004; Van der Heijden et al., 2009). We operationalize these cues in terms of the individual employee’s perception of OLC and propose that directional age differences become a less salient aspect in the prediction of supervisory ratings of employability when employees experience a supportive learning climate. Our third hypothesis, therefore, is as follows:

*Hypothesis 3: Greater perceived OLC will decrease the strength of the negative relationship between directional supervisor-subordinate age differences and supervisory employability ratings with respect to the subordinate’s: (a) Occupational Expertise, (b) Anticipation/Optimization, (c) Personal Flexibility, (d) Corporate Sense and (e) Balance*.

#### Training and Development

Participating in training and development opportunities, and being able to transfer this training into one’s work, is associated with higher levels of employability (Gerken et al., 2016; Van der Heijden et al., 2016; Bozionelos et al., 2020). For older workers, HRM policies and practices which encourage development are particularly important (Pak et al., 2019). Such investment also signals to employees that they are valued, enhancing career satisfaction and intention to remain (Armstrong-Stassen and Ursel, 2009). As indicated above, however, time spent on training and development diminishes significantly over 40 (Fleischmann et al., 2015), as the ‘pay-off period’ of the investment is expected to be shorter. This age effect seems to be stronger for long-term activities aimed at broadening knowledge and skills as supervisors tend to focus on employees’ current roles rather than on future employability (Van der Heijden et al., 2009). Evidence on human capital investment by late career workers themselves similarly suggests that they are less willing to participate in activities that provide primarily general or broader skills (Simpson et al., 2002; Veld et al., 2015) even though these are especially relevant for their career sustainability (Van der Heijden and De Vos, 2015; De Vos et al., 2020).

Regrettably, a lack of opportunity or willingness to continue development erodes capital invested in people and leads to lower levels of motivation, productivity, innovation and adaptation (Ybema et al., 2017; Sitzmann and Weinhardt, 2018), leaving employees vulnerable in insecure job markets. Moreover, age-related barriers in accessing training and development opportunities are actionable from a legal perspective (Fisher et al., 2017).

We acknowledge that training cannot be assumed to produce learning or transfer to one’s job performance. Individuals are frequently unable to utilize the knowledge gained from training courses due to factors such as the relevance to their current job, the extent to which training is perceived to be practical, and whether it is provided at the right time and through the right methods (Bell et al., 2017). Nevertheless, our focus here concerns the value of training for controlling the effects of directional age difference. When employees are enabled to continuously develop their professional knowledge and skills through access to job-relevant training, we propose that age norms which could influence supervisors with respect to their employees’ career potential are less relevant (Vickerstaff and Van der Horst, 2021) and that directional age differences are less salient with respect to employability ratings. We hypothesize, therefore, as follows:

*Hypothesis 4: Greater participation in training/development, and opportunities to apply these to one’s job, will decrease the strength of the negative relationship between directional supervisor-subordinate age difference and supervisory employability ratings with respect to the subordinate’s: (a) Occupational Expertise, (b) Anticipation/Optimization, (c) Personal Flexibility, (d) Corporate Sense and (e) Balance*.

## Materials and Methods

### Research Design and Sample

Data was gathered from supervisor-subordinate pairs working for a large Dutch subsidiary of a French multinational whose main products were glass and high-quality building materials. The company had actively encouraged employee participation in training and development programmes and supported career enhancement. In return for completing an online questionnaire for the study, respondents were told they would receive an anonymous, automated feedback report with recommendations on how to improve their employability based on their survey responses. In addition, full-day feedback workshops targeted at both employees and their supervisors were offered by the researchers. These incentives and the company’s positive approach to career enhancement may have positively influenced willingness to participate. Questionnaire data was collected on supervisors’ perceptions of subordinates’ employability, and employees’ perceptions of the company’s learning climate and training and development activities. In order to avoid overburdening supervisors, preserve data reliability, and ensure independence of data points, instructions asked each supervisor to complete employability ratings for a maximum of three employees, and if applicable divide these across age categories: young (20–34 years), middle-aged (35–49 years) and senior (50 years and older). We sought a sample which was a valid reflection of the distribution of respondents across departments, age groups, gender, and educational level. With very few exceptions, this appeared to be the case implying that we did not have to use multi-level techniques to account for the intra-group dimension of supervisor evaluations in analysis.

In the final sample, we included only employees with at least a high school education (to eliminate another potential influence on supervisor ratings). Only two had lower than middle school education and were dropped. We also eliminated supervisor-subordinate pairs with incomplete data. From 308 original pairs, analysis focused on a reduced number of 295 pairs (response rate 92%); *employees*: 254 males (84%), 41 females (16%); age (*M* = 41, *SD* = 9.15; 20–34 years (*n* = 73), 35–49 years (*n* = 153), 50 years or more (*n* = 69)); 79% with at least high school/equivalent qualifications, 21% with a higher degree; organizational tenure (*M* = 10.74 years; *SD* = 9.61); *supervisors*: 280 males (95%), 15 females (5%); mean age 43 (*SD* = 7.96). Forty-seven per cent of the supervisor-employee relationships had existed for 2 years or less; 34% had existed for three to 6 years; and 19% for 7 years or more.

### Measures

Directional age difference (Age Distance). A difference score was calculated by subtracting supervisor age from subordinate age. A difference score of 0 represents a subordinate and superior identical in age. Negative scores indicate a subordinate who is younger than their supervisor; positive scores indicate that the subordinate is older (Tsui and O’Reilly, 1989). In this sample, 40% of dyads represented positive scores with subordinates being older than their supervisors, and 57% of dyads represented negative scores with supervisors being older than their subordinates.

Employability was assessed with Van der Heijde and Van der Heijden’s (2006) thoroughly validated five scales, that meet the criteria for convergent and discriminant validity, measuring: (1) Occupational Expertise (15 items), (2) Anticipation/Optimization (8 items), (3) Personal Flexibility (8 items), (4) Corporate Sense (7 items), and (5) Balance (9 items). Supervisors were asked to indicate the employability of their subordinates. Example items for each dimension are: ‘By virtue of my experience with him/her, I consider him/her … competent to be of practical assistance to colleagues with questions about the approach to work’ (ranging from ‘not at all’ to ‘extremely’; Occupational Expertise); ‘(S)he is … focused on continuously developing him/herself’ (ranging from ‘not at all’ to ‘a considerable degree’; Anticipation/Optimization); ‘(S)he adapts to developments within the organization …’ (ranging from ‘very badly’ to ‘very well’; Personal Flexibility); ‘(S)he manages to exercise … influence within the organization’ (ranging from ‘very little’ to ‘a very great deal’; Corporate Sense); and ‘The time (s)he spends on his/her work and career development on the one hand and his/her personal development and relaxation on the other are … evenly balanced’ (ranging from ‘not at all’ to ‘a considerable degree’; Balance). All items were scored on a six-point scale. Cross-cultural research in seven European countries showed that Cronbach’s alpha ranged from 0.82 to 0.96, depending upon country, for Occupational Expertise, from 0.67 to 0.91 for Anticipation/Optimization, from 0.68 to 0.89 for Personal Flexibility, from 0.83 to 0.92 for Corporate Sense, and from 0.82 to 0.96 for Balance (Van der Heijden et al., 2005, 2009, 2018). Tests of scale reliability and validity, testing convergent, discriminant, and predictive validity (for career success) have yielded promising results (*ibid*; Van der Heijden et al., 2009, 2018).

Duration of the supervisory relationship was measured in the supervisors’ questionnaire with the following categories: 1 ‘< 1 year’, 2 ‘1–2 years’, 3 ‘3–4 years’, 4 ‘5–6 years’, 5 ‘7 years or longer’.

Organizational learning climate was measured as subordinate perceptions of the three dimensions of the validated Learning Climate Questionnaire (Bartram et al., 1993): (1) Time (10 items), (2) Team Style (10 items) and (3) Opportunity to Develop (10 items; see also Van der Heijden et al., 2018). All items were scored on a five-point rating scale ranging from 1 ‘never true’ to 5 ‘always true’. Example items were: ‘In some parts of the job there is not enough time to keep up with changes’ (reverse scored; Time); ‘If I have a question about my job there is someone available to answer it’ (Team Style); ‘I have opportunities to find out about issues outside my immediate job’ (Opportunity to Develop). Cross-cultural research in seven European countries showed that Cronbach’s alpha ranged from 0.67 to 0.85 for Time, 0.76 to 0.87 for Team Style, and 0.69 to 0.81 for Opportunities to Develop, depending upon country (Van der Heijden et al., 2005). Although the three dimensions have been shown to have good discriminant validity, principal components analysis found that all items loaded on a single factor which explained 70% of variance. Therefore, in the present study, a single composite variable (*α* = 0.78) representing each subordinate’s perceived OLC was created from the mean of all items in order to reduce the number of independent variables and increase the power of the regression analysis.

Training and development was measured with two variables which asked subordinates (Van der Heijden, 2002): (1) total number of days in the past year spent on training/development courses in either their current expertise/job area, adjacent expertise/job area, different or new expertise/job area, or personal development; and (2) whether they were able to apply knowledge and skills gained through training and development in their current jobs (measured as 1 ‘No or had not received training/development in that area’, 2 ‘yes, but not without some effort’ or 3 ‘yes, immediately and without any effort’).

Control variables: Employee age in years, gender, educational qualification, and organizational tenure (in months) were included as control variables. Gender was coded 1 for males and 2 for females. Highest level of educational qualification was measured by a single item on a scale from 1 ‘high-school or equivalent’ to 5 ‘doctorate (PhD)’ and recoded to either high school or equivalent (1) or higher degree (2).

### Analysis

The effect of directional age difference (Age Distance) on ratings of employability (Hypothesis 1) was examined using a two-step hierarchical regression analysis with control variables entered in the first step, and Age Distance in the second. Examination of scatter plots indicated a negative linear relationship between Age Distance and each of the five employability scales, suggesting that linear regression models were suitable for the data. The association between Age Distance and the moderators was moderate or negligible indicating no multi-collinearity between variables (Table 1). Hypotheses concerning the moderating effects of duration of supervisory relationship (Hypothesis 2), OLC (Hypothesis 3) and training and development (Hypothesis 4) were tested through hierarchical regression analyses examining the additional explained variance of each predictor and their interaction terms with Age Distance. First, the hypothesized moderators of the Age Distance-supervisor ratings relationship were added to the equation with control variables and Age Distance as a block to test main effects, followed by the interaction of each predictor with Age Distance.

**Table 1:** Means, standard deviations and inter-correlations between study variables.

	Variable	*M*	*SD*	1	2	3	4	5	6	7	8	9	10	11	12	13	14
1	Employee age	41.15	9.16														
2	Gender (1 = male, 2 = female)	1.17	0.37	−0.23													
3	Education (1 = high school, 2 = higher degree)	1.20	0.41	−0.21	0.06												
4	Organizational tenure (months)	129.11	115.38	0.57	−0.03	−0.22											
5	Age Distance (employee older)	−1.97	11.42	0.72	−0.08	−0.18	0.40										
6	Duration of supervisory relationship	3.02	1.29	0.02	0.00	−0.07	0.08	−0.40									
7	Organizational Learning Climate	3.39	0.44	−0.10	−0.01	0.10	−0.10	−0.21	0.14	**0.78**							
8	T&D days	18.65	52.48	−0.07	−0.03	0.10	−0.07	−0.03	−0.07	0.10							
9	Opportunity to apply training/current job	1.99	0.89	0.04	−0.13	0.15	−0.07	0.02	−0.07	0.24	0.26						
10	Occupational Expertise	4.40	0.68	−0.17	0.04	0.03	−0.05	−0.31	0.25	0.11	0.00	0.06	**0.95**				
11	Anticipation/Optimization	3.51	0.71	−0.29	−0.07	0.14	−0.21	−0.38	0.30	0.20	0.07	0.17	0.70	**0.89**			
12	Personal Flexibility	3.98	0.69	−0.35	−0.04	0.12	−0.35	−0.38	0.07	0.17	0.04	0.13	0.72	0.76	**0.88**		
13	Corporate Sense	3.94	0.71	−0.09	−0.09	0.07	−0.04	−0.26	0.24	0.14	0.01	0.14	0.77	0.70	0.72	**0.85**	
14	Balance	4.19	0.55	−0.14	−0.07	−0.07	−0.06	−0.19	0.15	0.18	0.10	0.16	0.60	0.56	0.59	0.51	**0.83**

Employee-supervisor pairs *N* = 295. T&D ‘Training & Development’. Entries on the diagonal are Cronbach’s alpha reliability coefficients. *r* ≥ 0.12 significant at *p* < 0.05; *r* ≥ 0.17 significant at *p* < 0.01; *r* ≥ 0.20 significant at *p* < 0.001.

## Results

Table 1 shows the means, standard deviations, reliability coefficients, and correlations between study variables. The OLC composite and five employability scales demonstrated good internal consistency above the 0.70 threshold normally recommended. The possibility of age stereotyping was suggested by the inverse relationships between employee age and all five supervisor ratings of employability. However, age also was inversely related to educational level (*r* = −21, *p* < 0.001) and positively related to tenure (*r* = 0.57, *p* < 0.001), suggesting that older employees were relatively less qualified and had lower organizational mobility. Mean Age Distance indicated that the majority of employees was younger than their supervisors (*M* = −1.97, *SD* = 11.42). Age Distance was significantly inversely related with supervisor ratings of Occupational Expertise (*r* = −0.31), Anticipation/Optimization (*r* = −0.38), Personal Flexibility (*r* = −0.38), Corporate Sense (*r* = −0.26), and Balance (*r* = −0.19), as well as with subordinates’ perceived OLC (*r* = −0.21). Number of days spent on training and development was unrelated to any of the employability dimensions. The opportunity to apply training and development was positively related to all employability dimensions, except for Occupational Expertise.

Directional age difference effect. Initial analysis supported the hypothesized relationship of Age Distance with all five employability dimensions (Hypothesis 1). Age Distance accounted for a significant incremental variance in ratings of Occupational Expertise (Δ*R*^2^ = 0.07, *F*(1,289) = 22.44, *p* < 0.001), Anticipation/Optimization (Δ*R*^2^ = 0.06, *F*(1,291) = 19.65, *p* < 0.001), Personal Flexibility (Δ*R*^2^ = 0.03, F(1,289) = 9.81, *p* < 0.01), Corporate Sense (Δ*R*^2^ = 0.07, F(1,291) = 21.11, *p* < 0.001) and Balance (Δ*R*^2^ = 0.01, F(1,291) = 4.26, *p* < 0.05) from the equation with control variables only (Table 2, Model 1). In each case, supervisory ratings were inversely related to Age Distance (Occupational Expertise, *β* = −0.38, *p* < 0.001; Anticipation/Optimization, *β* = −0.35, *p* < 0.001; Personal Flexibility, *β* = −0.24, *p* < 0.01; Corporate Sense, *β* = −0.37, *p* < 0.001; and Balance, *β* = −0.17, *p* < 0.05), suggesting that the older the subordinate relative to their supervisor, the more negatively they were rated by the supervisor on these dimensions.

**Table 2:** Hierarchical regressions for supervisory ratings of five dimensions of employability.

Predictor variables	DV: Occupational Expertise			DV: Anticipation/Optimization			DV: Personal Flexibility			DV: Corporate Sense			DV: Balance		
	*1*	*2*	*3*	*1*	*2*	*3*	*1*	*2*	*3*	*1*	*2*	*3*	*1*	*2*	*3*
Employee age	0.08	−0.03	−0.06	−0.02	−0.20^*^	−0.23^*^	−0.07	−0.09	−0.12	0.14	0.05	0.03	−0.07	−0.16	−0.20^*^
Gender (female)	0.03	0.02	0.02	−0.11	−0.11^*^	−0.12^*^	−0.09	−0.07	−0.08	−0.11	−0.09	−0.09	−0.11	−0.09	−0.09
Education (degree)	−0.01	−0.01	−0.02	0.07	0.06	0.04	0.02	0.00	−0.01	0.05	0.04	0.03	−0.11	−0.14^*^	−0.16^**^
Tenure (month)	0.06	0.06	0.04	−0.06	−0.05	−0.07	−0.21^**^	−0.21^**^	−0.22^**^	0.04	0.05	0.03	0.02	0.04	0.01
Age Distance (AD)	−0.38^***^	−0.24^**^	−0.20^*^	−0.35^***^	−0.11	−0.07	−0.24^**^	−0.23^*^	−0.18	−0.37^***^	−0.26^*^	−0.25^*^	−0.17^*^	−0.05	−0.01
Duration of relationship		0.15^*^	0.14^*^		0.26^***^	0.24^***^		−0.01	−0.03		0.11	0.10		0.10	0.07
OLC		0.03	0.02		0.08	0.08		0.08	0.07		0.03	0.02		0.12^*^	0.12^*^
T&D days		−0.02	−0.01		0.01	0.01		−0.03	−0.01		−0.02	−0.02		0.06	0.07
Opportunity to apply training		0.09	0.10		0.16^**^	0.17^**^		0.10	0.11		0.12	0.13^*^		0.14^*^	0.15^*^
AD × duration of relationship			−0.12^*^			−0.16^**^			−0.16^**^			−0.11			−0.22^***^
AD × OLC			0.06			0.03			0.06			0.06			0.05
AD × T&D days			0.02			−0.01			0.04			−0.03			0.03
AD × Opportunity to apply training			−0.01			0.01			−0.02			−0.01			−0.02
Adjusted *R*^2^	0.09	0.11	0.10	0.15	0.22	0.23	0.18	0.19	0.21	0.08	0.09	0.09	0.04	0.08	0.12
Δ*R*^2^	0.07	0.02	0.02	0.06	0.08	0.02	0.03	0.02	0.03	0.07	0.02	0.01	0.01	0.05	0.04
*F* change	22.44^***^	1.88ns	1.29	19.65^***^	7.21^***^	2.09ns	9.81^**^	1.639ns	2.48^*^	21.11^***^	1.78ns	1.015ns	4.26^*^	4.26^**^	3.69^**^
*df*	1,289	4,285	4,281	1,291	4,287	4,283	1,289	4,285	4,281	1,291	4,287	4,283	1,291	4,287	4,283

OLC, Organizational Learning Climate. Standardized regression coefficients. For Model 1, the change in R2 is calculated relative to equation with control variables only (equation not shown).

* *p* < 0.05,

** *p* < 0.01,

*** *p* < 0.001. ns, Not significant.

The addition of duration of supervisory relationship, perceived OLC, training/development days, and opportunity to apply training over and above control variables and Age Distance (Model 1) added a statistically significant increment to the adjusted *R*^2^ value for Anticipation/Optimization (Δ*R*^2^ = 0.08, *F*(4,287) = 7.21, *p* < 0.001) and Balance (Δ*R*^2^ = 0.05, *F*(4,287) = 4.26, *p* < 0.01; Table 2, Model 2). The previously significant Age Distance coefficients for these dependent variables become non-significant in Model 2 (*β* = −0.11 and *β* = −0.05 for predicting Anticipation/Optimization and Balance, respectively). In the equation for Anticipation/Optimization, duration of supervisory relationship (*β* = 0.26, p < 0.001) and the subordinates’ perception of opportunity to apply training (*β* = 0.16, p < 0.01) were positively related to supervisory ratings. In the equation for Balance, perceived OLC (*β* = 0.12, *p* < 0.05) and opportunity to apply training (*β* = 0.14, p < 0.05) were positively related to supervisory ratings. For the remaining dependent variables, where these additional variables did not improve *R*^2^, the negative relationship between Age Distance and supervisory ratings remained significant in Model 2 (Occupational Expertise, *β* = −0.24, *p* < 0.01; Personal Flexibility, *β* = −0.23, *p* < 0.05; and Corporate Sense, *β* = −0.26, *p* < 0.05). In the full model containing interaction terms (Model 3), Age Distance remained significant for only two dimensions (Occupational Expertise, *β* = −0.20, *p* < 0.05) and Corporate Sense (*β* = −0.25, *p* < 0.05). Thus, the findings provide partial support for Hypothesis 1 that higher Age Distance is associated with lower supervisory ratings for the subordinate regardless of the learning context, as this was found for some aspects of employability only.

**Figure 1:**
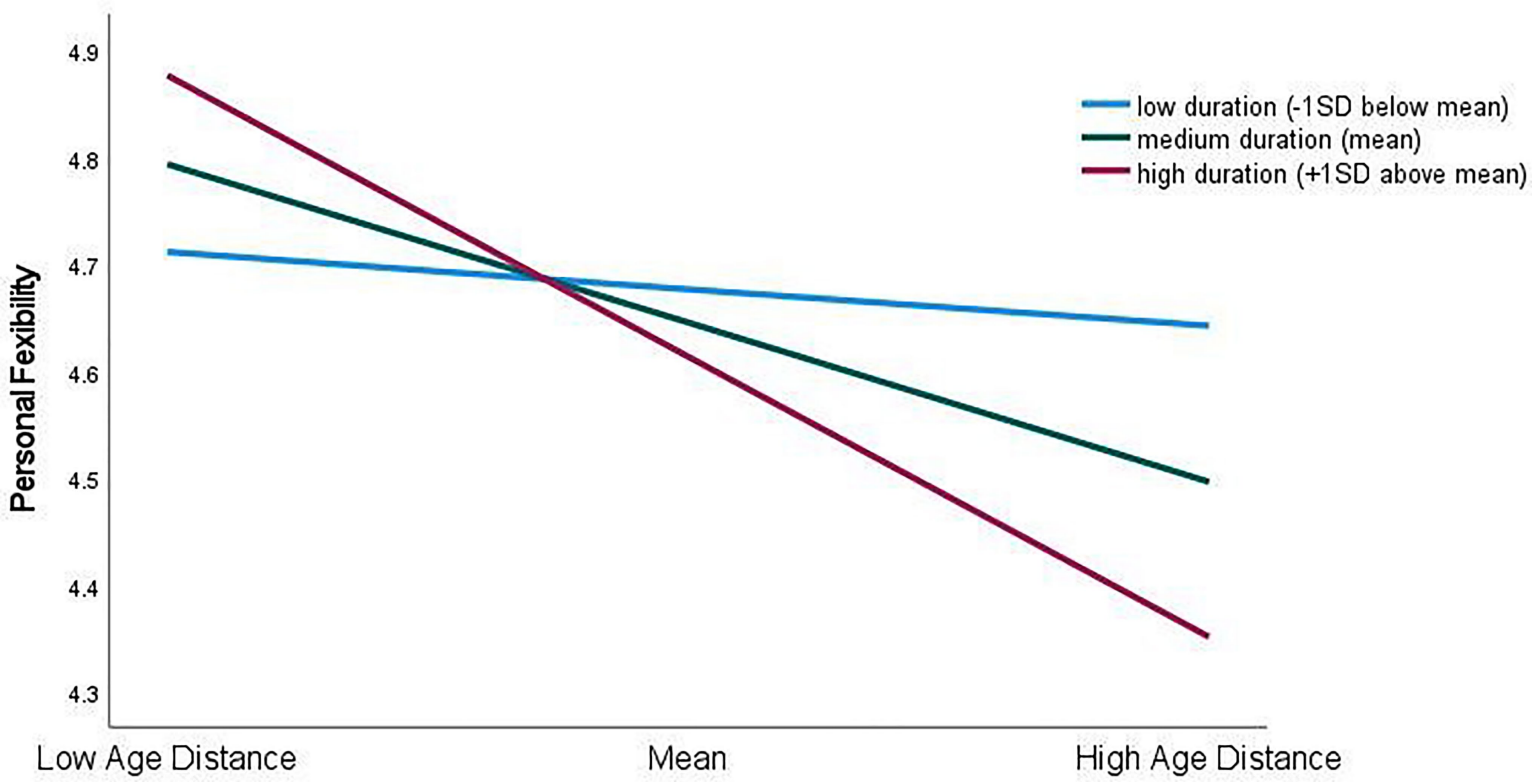
Interaction effects between Age Distance and duration of supervisory relationship for prediction of supervisory ratings of Personal Flexibility.

**Figure 2:**
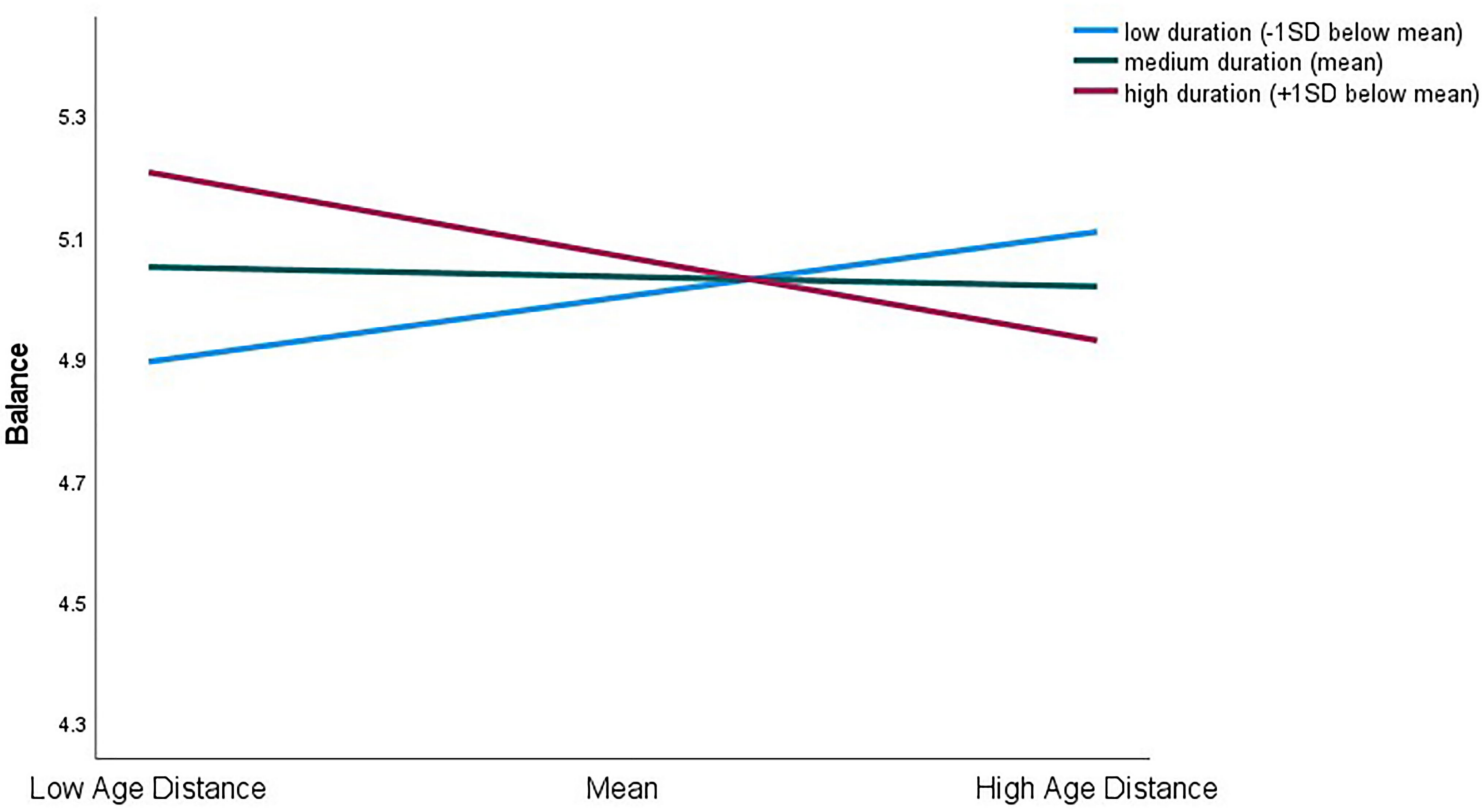
Interaction effects between Age Distance and duration of supervisory relationship for prediction of supervisory ratings of Balance.

Moderator effects. Model 3 in Table 2 shows that the interaction terms added significant incremental variance only for the prediction of Personal Flexibility (Δ*R*^2^ = 0.03, *F*(4,281) = 2.48, *p* < 0.05) and Balance (Δ*R*^2^ = 0.04, *F*(4,283) = 3.69, *p* < 0.01) and that duration of the supervisory relationship was the only significant moderator. However, the direction of the findings do not support Hypothesis 2 which expected a longer working relationship to decrease the strength of the negative relationship between Age Distance and the specific employability ratings. More specifically, the negative coefficients for the interaction terms (Personal Flexibility, *β* = −0.16, *p* < 0.01; Balance, *β* = −0.22, *p* < 0.001) and Figures 1, 2 show that, although supervisory ratings for all subordinates were generally high on the six-point rating scales, duration of dyadic tenure did not have a consistent effect.

Simple slopes’ tests for ratings of Personal Flexibility on Age Distance at different levels of dyadic tenure as represented in Figure 1 found that the coefficients for low (*β* = −0.05, *p* = 0.673) vs. medium (*β* = −0.21, *p* < 0.001) duration, and medium vs. high (*β* = −0.38, *p* < 0.001) duration were not significantly different at *p* < 0.05, however the coefficients for low vs. high duration were [*t*(586) = 2.11, *p* < 0.05]. Thus, Age Distance was not a significant predictor of ratings for low duration relationships, but there was a negative relationship between Age Distance and supervisory ratings in high duration relationships.

For ratings of Balance on Age Distance (Figure 2), high Age Distance was unexpectedly associated with higher (rather than lower) ratings in low duration relationships, while in high duration relationships, again, the relationship between Age Distance and balance ratings was negative. As well as reflecting relationships in entirely different directions, the coefficient for low duration relationships (*β* = 0.20, *p* < 0.01) was significantly different from that for high duration relationships (*β* = −0.25, *p* < 0.001, *t*(586) = 2.70, *p* < 0.01), but not significantly different from that for medium duration where the coefficient itself was also not significant (*β* = −0.03, *p* = 0.106).

The non-significant interaction terms (Age Distance × Duration of the relationship) in the equations for Occupational Expertise, Anticipation/Optimization and Corporate Sense, as well as the persistent significant coefficient for Age Distance for all five employability dimensions, highlights that in these cases, subordinates in low Age Distance relationships received higher ratings than those in high Age Distance relationships, regardless of relationship duration.

None of the interactions between Age Distance and OLC (Hypothesis 3) or training/development (Hypothesis 4) was statistically significant. Even though employees who perceived more opportunities to apply training tended to receive higher supervisor ratings for Anticipation/Optimization, and employees who perceived more positive OLC and opportunities to apply training tended to receive higher supervisor ratings for Balance (Table 2, Model 2), this positive learning context as perceived by subordinates did not buffer the negative relationship between Age Distance and supervisor ratings. Hypotheses 3 and 4, therefore, were not supported with our data.

## Discussion

This study considered (directional) age difference effects in the context of supervisory ratings of subordinates’ five competence-based employability dimensions (Van der Heijde and Van der Heijden, 2006; Van der Heijden et al., 2018). Hypothesis 1 was partially supported by our findings. The greater the directional age difference between a supervisor and subordinate (younger supervisor-older subordinate), the lower supervisory ratings on all five dimensions of employability, controlling for demographic variables. Some contrasts across dimensions were found. For ratings of Occupational Expertise, Personal Flexibility and Corporate Sense, the directional age difference effect was persistent regardless of subordinates’ perception of a positive learning context. For the prediction of ratings of Anticipation/Optimization and Balance, supportive learning context explained more variance in supervisor ratings than directional age difference. Our findings provide partial support for Hypothesis 1 that directional age difference is associated with lower supervisory ratings.

As regards possible moderation effects, our study indicates that the only significant effects were for duration of the supervisory relationship in the prediction of Personal Flexibility and Balance ratings. However, these were not in the direction we expected. The directional age difference effect was only shown to occur in high duration relationships where we had rather assumed that a high duration relationship would entail greater familiarity between the supervisor and subordinate, herewith buffering the negative effect. We also found that in long duration relationships, directional age difference was associated with lower supervisory ratings of both Personal Flexibility and Balance. That is to say, greater familiarity (as in a high duration relationship) made the status incongruence effect stronger rather than weaker as expected. Also contrary to our expectations, for all other employability dimensions, we found no buffering effects of any learning context variable representing organizational climate, training participation or the opportunity to apply training.

The study makes several contributions to understanding age dissimilarity and its implications for developing older workers’ career potential. First, by examining five dimensions of employability which have shown strong discriminant validity (Van der Heijde and Van der Heijden, 2006; Van der Heijden et al., 2018), we provide further confirmation of a broad status incongruence effect based on age dissimilarity which had only previously been shown for ratings of Occupational Expertise (Van der Heijden, 2018). Specifically, the current empirical work shows that the effect extends to wider conceptions of how supervisors judge employability beyond job-related expertise, particularly to generic competences of flexibility and organizational awareness (measured here as Corporate Sense).

Following the line of reasoning provided by the sustainable careers paradigm (De Vos et al., 2020), this is an important and alarming outcome. Distressingly, while one’s current performance and future career potential should be protected across the life-span, it seems that both elements are in danger. Only when supervisors and employees combine their strengths in safeguarding the employee’s expertise at work, allowing them to meet current and future challenges and to cope with changes in one’s current and possible future employers, can employees preserve their health, happiness and productivity until retirement age (*ibid.*). Given the fact that employability is a key indicator of success in current labor markets, this study shows the importance of being aware of age dissimilarity and its consequences in the workplace.

A second contribution is in how familiarity in supervisor-subordinate relationships may impact judgments of older workers. Van der Heijden (2018) had observed a similar strengthening of a directional age difference effect on ratings of Occupational Expertise with longer duration relationships, rather than the expected buffering effect of longer dyadic tenure. Although the present study did not find any such moderation effect for ratings of Occupational Expertise, its presence for Personal Flexibility and Balance demonstrated that supervisors’ judgments of familiar older subordinates extend to more personal attributes, such as ability to adapt to changing requirements (i.e., Personal Flexibility), and to cope with competing demands in one’s private and work life which may lead to conflicts between employers’ and one’s own needs (i.e., Balance).

The absence of an effect for Personal Flexibility and the unexpected positive effect for Balance in short-term relationships suggests that in these situations and under conditions of high age dissimilarity, supervisors with less familiarity of their employees may more conscientiously use individuating information (i.e., related to the individual’s personal characteristics) in their ratings. For supervisors in longer-term relationships, and thus with greater familiarity of their employees, age dissimilarity may lead to a decrease in analytic processing of individuating information thereby increasing the supervisor’s reliance on age stereotypes in making evaluations (Smith et al., 2006; Garcia-Marques et al., 2016; Van der Heijden, 2018). Thus, greater familiarity seemed to increase the potential for stereotyping rather than, as hypothesized, leading to greater affective attachment, hence buffering potential stereotyping.

As noted in Van der Heijden (2018), the findings relating to dyadic tenure show the complex role of familiarity in shaping supervisory judgments of older subordinates’ career potential. From our findings, we may conclude in particular that the supervisor’s judgement about the employee’s added value in terms of (a) their domain-specific knowledge and skills (Occupational Expertise), (b) their capacity to adapt easily to all kinds of changes in the internal and external labor market that do not pertain to their immediate job domain (Personal Flexibility), and (c) their ability to compromise between their own opposing work, career, and private interests or between their employer’s and their own interests (Balance), are more negatively affected the more the two parties differ in terms of age.

Finally, the finding that subordinates’ perceptions of a positive learning climate, training and development opportunities and application of training in one’s job had no moderating effects suggests that it may be hard to influence the direction of supervisors’ evaluation of older employees through contextual organizational factors such as provision of greater learning opportunities. We had hypothesized that a more supportive learning context would facilitate access to increasingly scarce resources, both for the older worker in enhancing their competence and for the supervisory task of evaluating an older subordinate, thus buffering potential age difference effects on ratings of employability. However, contrary to our expectation, we found that none of the learning context variables reduced the negative effect of age dissimilarity on any of the dimensions. From our study, we conclude that the effect of directional age difference and its potential for supervisors to follow age norms in their evaluations seems hard to combat (*cf.* Van der Heijden, 2018). Focused attention on the dangers of evaluation bias, for instance by means of supervisor training, remains important for monitoring this issue, and being able to enhance opportunities for older workers in the final phase of their career. At the same time, a possible alternative explanation for our unexpected findings regarding the assumed moderating effect of the learning context variables might lie in the supervisor’s behavior itself. More specifically, where there is a large age discrepancy between supervisor and subordinate, especially in such a direction that portrays status incongruence (supervisor being younger than his/her subordinate), the supervisor him/herself is less inclined to stimulate their subordinate’s participation in training and development opportunities (*cf.* Boerlijst, 1994; Knies et al., 2015). More empirical research is needed to better understand the underlying mechanisms in this regard.

Despite being unable to support these hypotheses, perceived OLC and opportunity to apply training were both associated with all four generic employability competences, as shown in the intercorrelations (Table 1) for these two variables, respectively, as follows: Anticipation/Optimisation (*r* = 0.20, *p* < 0.001; *r* = 0.17, *p* < 0.01), Personal Flexibility (*r* = 0.17, *p* < 0.01; *r* = 0.13, *p* < 0.05), Corporate Sense (both *r* = 0.14, *p* < 0.05), and Balance (*r* = 0.18, *p* < 0.01; *r* = 0.16, *p* < 0.05). One or both of the aforementioned variables also added significant explanatory power in the prediction of Anticipation/Optimisation and Balance (Table 2; Model 2). Given these direct effects, we argue, therefore, that there is still an important role for contextual learning support in facilitating these more future-oriented and personal competencies. Such dimensions capture older workers’ capacity to adapt in a sustainable way to changing job requirements when their current expertise becomes obsolete or needs to be renewed (see also Jeske et al., 2017; Schrimpf et al., 2021).

### Limitations and Future Research

Generalizability could be questioned given that the data is drawn from a single national culture, sector and company. For instance, other cultures or occupations are likely to hold different relational norms with respect to age; e.g., some cultures place greater value on the wisdom of elders, or in some industries, it may be common for supervisors to be younger or have less tenure than their subordinates. The company represented in the present study demonstrated a proactive approach to employee development which is typical of large employers with formalized HRM practices. As such, we argue that it provides a relevant source of data for testing hypotheses about supervisor-subordinate relations and age differences which are applicable to other similar organizations with an espoused concern for human resource development. Future research, however, should cross-validate the findings in different country/occupational settings.

We optimized our design by using appropriate control variables (Spector, 2019) identified from existing research on age stereotyping in supervisor evaluations of subordinates. Nevertheless, the nature of our data means that alternative explanations for the effects observed cannot be excluded. A longitudinal design is required to explore whether outcomes related to dyadic tenure represent a selection effect, as subordinates with higher employability ratings may be more likely to remain with their supervisors longer. It is also acknowledged that asking supervisors to select the subordinates they rated (to a maximum of three) and who should receive the corresponding questionnaire could introduce upward bias in levels of employability and perceptions. Given that the sample was stratified across age groups, gender and educational level, and therefore reflective of the wider organisation, it was viewed as unnecessary to account for intra-group variance in supervisor evaluations. Multi-level consideration of how supervisors evaluate subordinates differently within the same team could be considered in future research (Joshi et al., 2011). A final limitation is that we tested hypotheses separately for five dependent variables, each with multiple independent variables, which may increase the chance of alpha (Type I) error. Given that our aim was not to generate a single causal model for the prediction of supervisory ratings, but rather to identify theoretically-significant variables for different types of ratings, we did not correct the *p*-values for regression coefficients (e.g., using a Bonferroni adjustment; Shaffer, 1995).

These limitations are mitigated to some extent by our use of multi-source data from supervisors and subordinates to represent dependent and independent variables, respectively, which ensured that the key relationships of interest were not affected by response set consistencies common to single-source data (Arnulf et al., 2014). Moreover, the consideration of five dimensions of employability in this study provided a holistic view of potential age stereotyping, which is rare in the scholarly literature so far. The findings stressed the importance of this issue in light of one’s career sustainability over time, thus elucidating a real-world phenomenon which has been under-researched.

Several areas for future research emerge from our study. First, more work using multi-level research designs examining supervisor-employee dyads’ age differentials with multiple subordinates reporting to the same supervisor is also recommended in order to detect possible intra-group differences. Second, multiple sources of dissimilarity should be acknowledged as possibly impacting subjective judgments of employability. We demonstrated, for instance, that long duration supervisory relationships do not imply high quality leader-member exchange, therefore, including measures of the quality of the relationship (Graen and Uhl-Bien, 1995) may be an important indicator of potential dissimilarity effects. Equally, future research should aim to detect alternative moderator variables that may counteract negative effects of age dissimilarity. Other sources of dissimilarity, such as personality, may offer further insights on potential stereotyping effects (e.g., Antonioni and Park, 2001). Measuring both perceived as well as actual age (dis)similarity as a predictor of supervisory evaluations in high and low age distance contexts also will enhance understanding of demographic (dis)similarity effects given the importance of perceptual measures in predicting inclusion outcomes, such as belongingness (e.g., Riordan and Wayne, 2008; Shore et al., 2011). Finally, although the chosen approach is justified given our preliminary analyses investigating the character of the data, polynomial regression models could be used to comply with some conceptual and methodological issues that are inherent to the practice of using difference scores (Edwards, 2002; Shanock et al., 2010).

### Practical Implications

Demographic and workplace trends signal the need to motivate and develop multiple generations particularly given the increasing importance of employee resilience in adapting to digital transformation (Carbonero and Scicchitano, 2021; Trenerry et al., 2021). Employers’ growing emphasis on adaptability to digitalization means that status incongruence resulting from age (dis)similarity may become an even more problematic source of age stereotyping in performance appraisal. In addition, more merit-based rather than seniority-based promotion systems make being supervised by younger managers more likely (Cappeli and Novelli, 2010) and a potential threat for older workers’ engagement (Kulik et al., 2016).

Our findings suggest that older workers’ employment opportunities may be hindered due to negative supervisor perceptions of workers’ Occupational Expertise, Personal Flexibility and Corporate Sense. These dimensions of employability reflect both role and extra-role performance, and the presence of more employee developmental opportunities did not buffer these effects in this study. Combining supervisor appraisals and self-assessments and making potential rating biases fully transparent may be an important first step. Interventions such as diversity or unconscious bias training of supervisors have become popular, but the implications of age (dis)similarity for unfamiliar and familiar supervisor-subordinate pairs is less well-known (Kulik et al., 2016). Obviously, such a self-appraisal process is largely dependent on the psychometric qualities of the specific measures used, thus requiring well validated tools to capture supervisor attitudes and potential age stereotyping (for an example see Rego et al., 2017).

The study also found that providing opportunities for further development of career potential (through a developmental learning climate and opportunity to apply one’s job-specific training) were associated with higher supervisory ratings of Anticipation/Optimization, Personal Flexibility, Corporate Sense, and Balance regardless of age difference. These findings confirm the value of providing learning opportunities to allow workers of any age to prepare for future changes and to adapt their skills to changing job requirements (Anticipation/Optimization), to adapt to all kinds of changes in the internal and external labor market, and to increase organizational awareness (Corporate Sense) while avoiding the potentially negative effects of conflicting employer demands for personal wellbeing (hence maintaining Balance). The latter negative effects in particular have been identified with skill-enhancing HRM practices like training (Ogbonnaya and Messersmith, 2019). More broadly, accurate understanding of staff employability across age groups is not only ethical, but also essential for the adaptability and performance of the organization itself (Stoffers and Van der Heijden, 2009).

## Conclusion

Staying employable across one’s career is critical to extending working life until, and possibly even after, retirement age. Our study, however, indicates that supervisor-subordinate age dissimilarity where supervisors are younger than their subordinates is a threat to how older employees are evaluated, and as a result, their career sustainability. Approaching employability from the perspective of relational demography, we advanced on previous studies of age stereotyping by incorporating an important contextual factor for maintaining sustainable careers - the employee’s supervisor as a key stakeholder (Van der Heijden and De Vos, 2015). Political organizational context and supervisors’ own motivations have been shown to play a role in shaping evaluations. For example, supervisor ratings of their employee’s extra-role behavior has been shown to be consistent with the employee’s in-role performance in low political contexts, while in highly political contexts, ratings will be more likely to reflect the supervisors’ self-interest and to be unaffected by levels of in-role performance (Rosen et al., 2017). We add to this evidence by showing potential supervisor stereotyping demonstrated by directional age differences on ratings of employability related to both in-role and extra-role performance. Wider developmental opportunities in the organization could not buffer this effect, while longer dyadic tenure increased it. Given the crucial role played by line managers in HRM implementation and employee development (Gilbert et al., 2011; Katou et al., 2021), this strong association between age dissimilarity and supervisory ratings of multiple dimensions of employability is all the more distressing.

## Data Availability Statement

The datasets presented in this article are not readily available because the data are not publicly available due to privacy or ethical restrictions. The data are available upon reasonable request from the authors. Requests to access the datasets should be directed to BH, beatrice.vanderheijden@ru.nl.

## Ethics Statement

The studies involving human participants were reviewed and approved by the Dutch Research Council. The participants provided their written informed consent to participate in this study.

## Author Contributions

All authors contributed equally to all aspects of preparation of this manuscript. BH led the data gathering for the study.

## Funding

This work was supported by the Nederlandse Organisatie voor Wetenschappelijk Onderzoek (Netherlands Organization for Scientific Research).

## Conflict of Interest

The authors declare that the research was conducted in the absence of any commercial or financial relationships that could be construed as a potential conflict of interest.

## Publisher’s Note

All claims expressed in this article are solely those of the authors and do not necessarily represent those of their affiliated organizations, or those of the publisher, the editors and the reviewers. Any product that may be evaluated in this article, or claim that may be made by its manufacturer, is not guaranteed or endorsed by the publisher.
